# *In Silico* Models of DNA Damage and Repair in Proton Treatment Planning: A Proof of Concept

**DOI:** 10.1038/s41598-019-56258-5

**Published:** 2019-12-27

**Authors:** Edward A. K. Smith, N. T. Henthorn, J. W. Warmenhoven, S. P. Ingram, A. H. Aitkenhead, J. C. Richardson, P. Sitch, A. L. Chadwick, T. S. A. Underwood, M. J. Merchant, N. G. Burnet, N. F. Kirkby, K. J. Kirkby, R. I. Mackay

**Affiliations:** 1 Division of Cancer Sciences, School of Medical Sciences, Faculty of Biology, Medicine and Health, The University of Manchester, Manchester, UK; 2 Christie Medical Physics and Engineering, The Christie NHS Foundation Trust, Manchester, UK; 3 The Christie NHS Foundation Trust, Manchester Academic Health Science Centre, Manchester, UK

**Keywords:** DNA damage and repair, Double-strand DNA breaks, Cancer

## Abstract

There is strong *in vitro* cell survival evidence that the relative biological effectiveness (RBE) of protons is variable, with dependence on factors such as linear energy transfer (LET) and dose. This is coupled with the growing *in vivo* evidence, from post-treatment image change analysis, of a variable RBE. Despite this, a constant RBE of 1.1 is still applied as a standard in proton therapy. However, there is a building clinical interest in incorporating a variable RBE. Recently, correlations summarising Monte Carlo-based mechanistic models of DNA damage and repair with absorbed dose and LET have been published as the Manchester mechanistic (MM) model. These correlations offer an alternative path to variable RBE compared to the more standard phenomenological models. In this proof of concept work, these correlations have been extended to acquire RBE-weighted dose distributions and calculated, along with other RBE models, on a treatment plan. The phenomenological and mechanistic models for RBE have been shown to produce comparable results with some differences in magnitude and relative distribution. The mechanistic model found a large RBE for misrepair, which phenomenological models are unable to do. The potential of the MM model to predict multiple endpoints presents a clear advantage over phenomenological models.

## Introduction

The primary advantage of proton beam therapy (PBT) over photon therapy is found in the apparent superior dose distribution delivered to the patient where the same target dose is delivered with a dose reduction to the surrounding healthy tissue^1^. Currently, total patient numbers treated by PBT are relatively small compared to the total for photon treatments and, consequently, the wealth of clinical knowledge of the biological effect of radiation is for photon absorbed dose. The translation of this knowledge to protons would be simple if the absorbed dose of photons and protons had equal biological effect. However, there is a known difference in the biological effect of the two radiation qualities^2^, and thus this translation is not trivial. To translate photon dose into proton dose, the concept of Relative Biological Effect (RBE) is commonly used:

1 $$RBE\mathop{=}\limits^{\Delta }{\left(\frac{{D}_{photons}}{{D}_{protons}}\right)}_{x}$$

where ${D}_{proton}$ is the proton dose required to obtain the same endpoint, $x$, as the reference photon dose, ${D}_{photon}$. There is no formally defined reference radiation quality, although experiments typically use a photon energy spectrum of a nominal 250 kV or Cobalt-60 source.

*In vitro* experiments reported in the literature demonstrate a dependence of RBE on many factors such as dose, linear energy transfer (LET), oxygenation, tissue type and biological endpoint^2^. However, the variability in the ensemble of *in vitro* data is too great to obtain a robust value of RBE across all of these factors. Instead, a constant RBE of 1.1 is applied to the absorbed dose in proton therapy treatment plans^3^. At present, there is no evidence demonstrating an adverse symptomatic effect. The use of this constant value of RBE means for the same dose, protons are assumed to be 10% more effective than photons across all parameters.

In recent years, further *in vitro* data has been published making the variation in RBE harder to ignore^4^. Nonetheless, the issues with the translation between *in vitro* and *in vivo* must be thought of when considering this data. Clinically, there is little evidence to suggest that the application of a constant RBE of 1.1 has a detrimental effect on patient outcome. However, studies outlining image changes in healthy tissue^5,6^ are beginning to challenge this argument along with evidence that the use of a constant RBE could produce sub-optimal treatment plans causing degradation in clinical effect^7^. There is a growing awareness within the community that variable RBE should be considered in the treatment planning process. However, the optimal method of doing so is not clear and stringent evidence that safety of treatments will not be compromised is required.

Given these uncertainties, the incorporation of LET has been proposed as an intermediate step^8^ to the full implementation of variable RBE into clinical plans. LET is a commonly used parameter to describe a particle’s track structure and is defined as the amount of energy deposited per unit length along a charged particle’s path^9^. There is strong* in vitro* evidence linking LET to biological effect and, as a physical parameter, LET can be calculated to a high degree of accuracy compared to biological parameters^2^. Furthermore, the clinical evidence^5,6^ of variable RBE suggests a link between the increased biological effect (i.e. image change) at the end of proton range with regions of elevated LET.

Approaches to incorporate LET into clinical plans have been varied. Some authors have explored the use of combinations of dose and LET, such as L*D^10^ or $D(1+\kappa L)$^10,11^ where L is dose-averaged LET ($LE{T}_{d}$), D is absorbed dose and $\kappa$ is a fitting parameter. However, uptake in the clinic has been slow due to a lack of consensus and concerns of the uncertainties in the appropriate way to combine LET and dose and how to determine appropriate fitting parameters. There is also a paucity of evidence demonstrating symptomatic toxicity through the lack of use of such models in treatment planning. Also, L*D has been shown to have a poor fit with data^10^.

Others have modelled RBE using phenomenological methods^12–15^, despite the restrictive uncertainty in the experimental data^2^. Generally, these models use parameters of dose, LET and the tissue-specific parameter, $\alpha$/$\beta$. Again, their uptake in the clinic has been limited due to uncertainty in the fitting data and thus how reliable the models are in predicting clinical effect. The experimental data used to fit these models is sometimes from non-human cell lines, and the phenomenological nature of the models makes it difficult to apply them to other endpoints or situations.

An alternative to these approaches is mechanistic modelling. There are several models of this type in the literature with varying complexity in how radiation damage and repair of DNA double-strand breaks (DSBs) are modelled^16–18^. Recently, a suite of mechanistic models to examine the DNA damage resulting from different radiation qualities has been developed^19,20^. These models have been used to investigate some of the factors surrounding RBE by simulating the effect of different radiation qualities on combined DNA and cell geometries in the Monte Carlo (MC) toolkit Geant4-DNA^21,22^. The structure and pattern of energy deposition on the DNA from these simulations is recorded and passed to the DNA Mechanistic Repair Simulator (DaMaRiS). Here, predictions are made on the efficacy and fidelity of repair at various time points up to 24 hours after radiation.

Previously published work using this model^19,20^ has demonstrated an increase in complex damage and DSBs in proximity to one another, with increasing LET. These breaks in close proximity were then predicted by the model to lead to an increased probability of incorrect DNA repair^20^. A series of simple correlations were established with inputs of absorbed dose and LET and outputs of endpoints of predicted yields of residual and misrepaired DSBs. These correlations allow for the accurate reproduction of the detailed model results. In this paper, the model compromising of both the damage and repair processes will be referred to as the Manchester Mechanistic (MM) model.

The work in this paper applies these previous findings into clinical treatment planning as a ‘proof of concept’ to demonstrate the potential of such an approach. GATE^23^, a framework for the MC toolkit Geant4^24^, has been used to calculate the absorbed dose and LET in each voxel of a proton therapy plan. Using the previously published correlations, we present maps showing predicted yields of residual and misrepaired DSBs in cells at each voxel. In principle, these maps may provide the clinician with additional valuable information on expected biological outcomes, allowing for identification of regions of heightened biological effect for differing endpoints.

## Methods

### Workflow for the variable RBE calculation in a treatment plan

This study is a retrospective analysis of a patient treatment plan. The patient gave informed consent for their data to be used for this purpose, and all data was handled according to GDPR regulations. The research was approved by the Radiotherapy Related Research committee at The Christie.

The patient presented in the case study is a 24-year-old female ependymoma patient treated with passively scattered proton beam therapy (PSPBT). An ependymoma case was selected as these patients are considered particularly at risk of severe toxicity, such as brainstem necrosis, if RBE values are above 1.1^25^. For the purpose of this study, a spot-scanning treatment plan was created at The Christie according to the protocol for this tumour site. A three-field beam arrangement (two lateral and one superior) Single Field Uniform Dose (SFUD) treatment plan with 1.8 Gy per fraction was created using a Varian ProBeam beam model in Eclipse${}^{TM}$ (v13.7, Varian, Palo Alto, USA) treatment planning system (TPS). The target and organs at risk (OARs) volumes were given clinician approval. The plan was assessed separately for robustness under a range uncertainty of 3.5% and under 3 mm shifts of the patient in the x, y, and z coordinates.

The patient presented in the case study is a 24-year-old female ependymoma patient treated with passively scattered proton beam therapy (PSPBT). An ependymoma case was selected as these patients are considered particularly at risk of severe toxicity, such as brainstem necrosis, if RBE values are above 1.1^25^.

For the purpose of this study, a spot-scanning treatment plan was created at The Christie according to the protocol for this tumour site. A three-field beam arrangement (two lateral and one superior) Single Field Uniform Dose (SFUD) treatment plan with 1.8 Gy per fraction was created using a Varian ProBeam beam model in Eclipse${}^{TM}$ (v13.7, Varian, Palo Alto, USA) treatment planning system (TPS). The lateral beam energy range was 79–121 MeV and the superior beam energy range was 115–145 MeV. The target and organs at risk (OARs) volumes were given clinician approval. The plan was assessed separately for robustness under a range uncertainty of 3.5% and 3 mm shifts of the patient in the x, y, and z coordinates.

AUTOMC (v20180613) is an in-house MC dose calculator built in Octave (v4.2.2). The basic process of the software is to translate the DICOM RT files of an Eclipse-made proton therapy plan into .mac text files. The software then drives the GATE (v8.1)^23^ / Geant4 (v10.3.3)^24^ environment and a computational cluster system to obtain a MC dose calculation before performing gamma analysis between the MC and TPS dose. The combination of the GATE and GEANT4 versions is known as GATE-RTion v1.0 which is constructed and dedicated for clinical use in light ion beam therapy. Its main role is to form part of the dosimetric verification of proton therapy plans at The Christie. For this work, AUTOMC was used to calculate absorbed dose and LET distributions for the patient plan.

GATE^23^ is a framework designed to aid medical physics simulations in the MC toolkit Geant4^24^. For this work, absorbed dose to water, $LE{T}_{t}$ to water and $LE{T}_{d}$ to water was calculated in 2 mm voxels using the QGSP_BIC physics list. Cuts of 0.1 mm were used for gamma, electron and positron radiation while cuts of 1 mm were applied for protons. The QGSP_BIC physics list has been previously shown to match other well-established physics lists used for proton therapy applications^26^. A beam model representative of a Varian ProBeam delivery system was used. The in-built LET scorer calculated $LE{T}_{t}$ and $LE{T}_{d}$ using the Geant4 method ‘GetElectronicStoppingPowerDEDX’. This method is insensitive to different initial MC parameters, which is particularly important for $LE{T}_{d}$^27,28^. The number of histories was scaled to achieve an approximate uncertainty of 1% Gy${}_{RBE=1.1}$ within the high dose region.

The MC-simulated absorbed dose and LET were then used as inputs to different biological models calculated within the $MATLA{B}^{TM}$ (R2017A Mathworks Inc., USA) environment. The models were also evaluated and visualised within this environment.

### LET${}_{d}$ and LET${}_{t}$

The following definitions of averaged LET were used in the variable RBE models in this paper:

$LE{T}_{d}$: The LET from each particle is weighted with respect to its contribution to local dose in each voxel, obtaining Eq. 2:

2 $$LE{T}_{d}={\sum }_{i}^{N}\frac{\Delta {E}_{i}}{{\Sigma }_{i}\Delta {E}_{i}}\frac{\Delta {E}_{i}}{\Delta {l}_{i}}$$

where $N$ is the total number of particles within the voxel, $\Delta {E}_{i}$ is the energy deposited by the $i$th particle in the voxel (keV) and $\Delta {l}_{i}$ is the path length of the $i$th particle ($\mu$m).

$LE{T}_{t}$: The LET from each particle is weighted with respect to its step length in each voxel, obtaining Eq. 3:

3 $$LE{T}_{t}={\sum }_{i}^{N}\frac{\Delta {l}_{i}}{{\Sigma }_{i}\Delta {l}_{i}}\frac{\Delta {E}_{i}}{\Delta {l}_{i}}$$

where $N$ is the total number of particles within the voxel, $\Delta {E}_{i}$ is the energy deposited by the $i$th particle in the voxel (keV) and $\Delta {l}_{i}$ is the path length of the $i$th particle ($\mu$m). Track-averaged LET is also known as the fluence-averaged LET.

### Biological models

The following models were used to calculate RBE-weighted dose using the MC-calculated absorbed dose and LET:

#### $LE{T}_{d}$ - weighted Dose Model

Several authors have applied this weighted dose model^10,29^. Its form is derived from the linear quadratic (LQ) model by assuming the concept of biologically effective dose will result in Eq. 4:

4 $$Dos{e}_{w}=D\cdot (1+\kappa \cdot LE{T}_{d})$$

where $Dos{e}_{w}$ is the $LE{T}_{d}$ - weighted dose, $D$ is the proton absorbed dose, $LE{T}_{d}$ is the dose-averaged LET and $\kappa$ is a fitting parameter. A $\kappa$ value of 0.055 $\mu m\hspace{2.22144pt}ke{V}^{-1}$ (shown in Table 1) was obtained by minimising the biological uncertainties of *in vitro* experimental data^10^.

**Table 1:** Shows the parameter values and standard error, as a percentage, (if available) for Eqs. 4, 5, 6, 7 and 8. ${Z}_{1}$, ${Z}_{2}$, ${Z}_{3}$ and ${Z}_{4}$ are taken from the McNamara^14^, $\kappa$ is taken from McMahon^10^ and a, b, c, d, e, f, g and h are taken from Henthorn^20^.

Parameter	Value $$\pm $$$$ \% $$	Unit
${Z}_{1}$	0.99064 $\pm$ 1.4	—
${Z}_{2}$	0.35605 $\pm$ 4.2	$Gy\ \mu m\ ke{V}^{-1}$
${Z}_{3}$	1.1012 $\pm$ 0.5	—
${Z}_{4}$	0.00387 $\pm$ 23.6	$G{y}^{-\frac{1}{2}}$
$\kappa$	0.0055	$\mu m\ ke{V}^{-1}$
a	0.1966 $\pm$ 0.4	—
b	0.008 $\pm$ 3.4	—
c	0.0736 $\pm$ 0.2	—
d	1.149 $\pm$ 1.0	$\mu m\ ke{V}^{-1}\ G{y}^{-1}$
e	24.1 $\pm$ 0.6	$G{y}^{-1}$
f	4.879E-4 $\pm$ 0.8	$\mu {m}^{2}\ ke{V}^{-2}$
g	2.84E-3 $\pm$ 4.7	$\mu m\ ke{V}^{-1}$
h	5.13E-2 $\pm$ 1.6	—
${\gamma }_{r}$	1.726 $\pm$ 2.0	$G{y}^{-1}$
${\gamma }_{m}$	0.0427 $\pm$ 16.7	$G{y}^{-1}$

#### McNamara Model^14^

This phenomenological model of RBE for PBT is based on the linear quadratic (LQ) model. The model was derived via a nonlinear regression fit to *in vitro* experimental data. The RBE-weighted dose is obtained via Eq. 5:

5 $$Dos{e}_{Mc}=D\ \cdot \left(\frac{1}{2D}\ \cdot \sqrt{{\left(\frac{\alpha }{\beta }\right)}_{x}^{2}+4D\ \cdot {\left(\frac{\alpha }{\beta }\right)}_{x}\cdot \left({Z}_{1}+\frac{{Z}_{2}}{{\left(\frac{\alpha }{\beta }\right)}_{x}}\cdot LE{T}_{d}\right)+4{D}^{2}\ \cdot {\left({Z}_{3}-{Z}_{4}\cdot \sqrt{{\left(\frac{\alpha }{\beta }\right)}_{x}}\right)}^{2}}-{\left(\frac{\alpha }{\beta }\right)}_{x}\right)$$

where $D$ is the proton absorbed dose (Gy), ($\alpha /\beta$)${}_{x}$ is the tissue-specific parameter of tissue $x$ (Gy), $LE{T}_{d}$ is the dose-averaged LET ($keV\hspace{2.22144pt}\mu {m}^{-1}$) and ${Z}_{1}$–${Z}_{4}$ are the parameters derived by fitting to cell survival data^14^ (shown in Table 1).

#### Manchester mechanistic model

The MM model^19,20^ explicitly considers the radiation damage to DNA and one of the primary repair pathways of DSBs, namely Non-Homologous End Joining (NHEJ). In both the damage and repair parts of this model, the various mechanistic components of the process have been fitted to biological data found in the literature. This process has been simulated in the Monte Carlo (MC) toolkit Geant4-DNA^21,22^.

The model starts with irradiating a spherical cell with a range of doses and LET (if a charged particle) of specified radiation type. The resulting energy depositions made by the radiation, directly and indirectly, are then re-simulated onto DNA strands and the probability distribution of break type is created with dependence on both dose and LET. The resulting pattern and geometry of DSBs is then passed to the repair portion of the model, named DaMaRiS.

DaMaRiS simulates the two broken ends of DSBs as they undergo sub-diffusion in the cell nucleus. During sub-diffusion, the attachment sequence of repair proteins necessary for DSB repair, via NHEJ, is given a chance to occur. After 24 hours, this process either results in a misrepaired, residual or fully repaired DSB. Residual refers to DNA damage which has not been repaired after the 24 hour period and misrepair refers to DSBs which has been incorrectly repaired. All cells are assumed to be in the G1 phase where NHEJ is dominant^30^.

The MM model is simulated at a cellular level and therefore is not suited for geometries at the scales relevant to clinic application. Instead, simple correlations have been fitted to the results from the MM model so residual and misrepaired DSB yields can calculated on a patient geometry. These correlations take inputs of absorbed dose and LETt and have been shown to be in good agreement to the full MM model in previously published work^20^. It should be noted the a-h parameters in these models do not have a physical meaning.

These correlations have been applied with Eq. 1 to obtain RBE-weighted dose for endpoints of residual yield ($Dos{e}_{r{\rm{\& }}m}$), misrepair yield ($Dos{e}_{m}$) and combined yield ($Dos{e}_{r{\rm{\& }}m}$). This is shown below in Eqs. 6, 7 and 8 where the yields for protons and photons for the specified endpoint are the numerator and denominator, respectively.

6 $$Dos{e}_{r}=D\cdot \frac{(d\cdot LE{T}_{t}+e)\cdot c}{{\gamma }_{r}}=D\cdot RB{E}_{r}$$

7 $$Dos{e}_{m}=D\cdot \frac{(d\cdot LE{T}_{t}+e)\cdot (a\cdot (\,f\cdot LE{T}_{t}^{2}+g\cdot LE{T}_{t}+h)+b)\cdot (1-c)}{{\gamma }_{m}}=D\cdot RB{E}_{m}$$

8 $$Dos{e}_{r{\rm{\& }}m}=D\cdot \frac{(d\cdot LE{T}_{t}+e)\cdot c+(d\cdot LE{T}_{t}+e)\cdot (a\cdot (f\cdot LE{T}_{t}^{2}+g\cdot LE{T}_{t}+h)+b)\cdot (1-c)}{{\gamma }_{r}+{\gamma }_{m}}=D\cdot RB{E}_{r{\rm{\& }}m}$$

where $D$ is absorbed dose (Gy), $LE{T}_{t}$ is track-averaged LET (keV $\mu$m${}^{-1}$) and a, b, c, d, e, f, g, and h are parameters derived to fit the DaMaRiS model to experimental data for endpoints of residual and misrepaired DSB yields. Parameters ${\gamma }_{r}$ and ${\gamma }_{m}$ are the average yields of residual and misrepair DSBs respectively, per Gy of Cobalt-60 photon irradiation. Values for a, b, c, d, e, f, g, h, ${\gamma }_{r}$ and ${\gamma }_{m}$ are shown in Table 1.

Equation 8 combines the end points of both residual and misrepaired DSB yields. By doing so, an assumption is made that these yields equally contribute to biological effect. It may also be assumed that all residuals and misrepaired DSBs present after 24 hours will result in cell kill.

## Results

**Figure 1:**
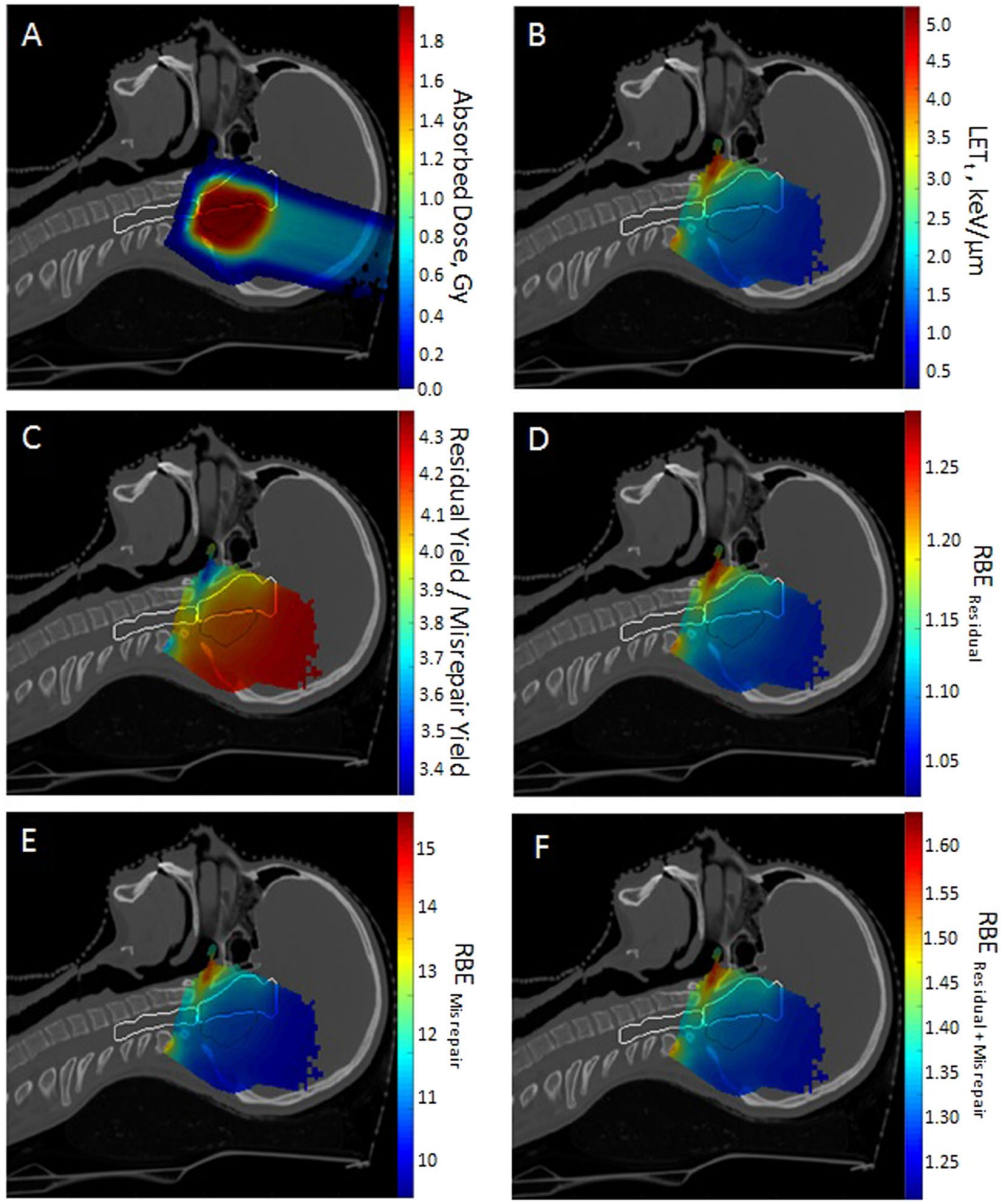
Calculation maps for the three field SFUD ependymoma case. (**A**) Absorbed dose (Gy). (**B**) Track-averaged LET (keV/$\mu$m). (**C**) Ratio of predicted average residual DSBs per cell against predicted average misrepaired DSBs per cell using previously published correlations^20^. (**D**–**F**) Relative Biological Effectiveness (RBE) maps of yields of residual DSBs, misrepaired DSBs and combined misrepair and residual DSBs. (**D**) Map of $RB{E}_{r}$ (Eq. 6). (**E**) Map of $RB{E}_{m}$ (Eq. 7). (**F**) Map of $RB{E}_{r{\rm{\& }}m}$ (Eq. 8). Critical organs contours (white) and the CTV contours (black) are shown.

Figure 1 presents a range of distributions calculated for the SFUD plan of the ependymoma case with a central sagittal CT slice shown. Figure 1A shows the absorbed dose to water and Fig. 1B shows the $LE{T}_{t}$ to water. $LE{T}_{t}$ is elevated in the posterior wall of the nasopharynx and, to a lesser extent, the anterior brainstem. This elevation is expected in these regions as they coincide with the overlap of the distal or lateral edges of the three fields. It is known these parts of the fields have higher LET values^31^.

Figure 1C shows the ratio of predicted residual DSBs yield and misrepaired DSBs yield calculated by the MM model. This demonstrates the yield of residual DSBs is always greater than the yield of misrepair DSBs. However, this ratio decreases at the distal edge of the beams corresponding to a relative increase in misrepair. This decrease is expected as misrepair has a greater dependence on $LE{T}_{t}$ and therefore more closely follows the range increase of LET in comparison to the residual yield.

Figure 1D–F shows the spatial distributions of RBE for residual DSBs ($RB{E}_{r}$), misrepaired DSBs ($RB{E}_{m}$) and combined misrepair and residual DSBs ($RB{E}_{r{\rm{\& }}m}$) for the three field SFUD plan of the ependymoma case. These maps follow Eq. 1 and compare the specified endpoint (residual, misrepair and residual + misrepair) between protons and photons for a given dose using Eqs. 6,7 and 8. The relative distributions of the different RBE endpoints are broadly similar with the main differences being the magnitude of RBE values and a slightly greater distal expansion for the misrepair RBE.

$RB{E}_{r}$ ranges from 1.1–1.3 across the planned target volume (PTV) and critical structures. Small regions outside of these structures fall slightly below 1.1 to a minimum of 1.03. $RB{E}_{m}$ is considerably larger with values ranging between 10 and 16 in the ROIs. These very high RBE values are due to the relatively low yield of misrepaired DSBs in photon radiation. This suggests that this is a mode of damage which occurs more frequently under proton irradiation than in photons. The endpoint of $RB{E}_{r{\rm{\& }}m}$ is the sum of both misrepair and residual yields. As the yield of misrepair is low in comparison to the yield of residual, $RB{E}_{r{\rm{\& }}m}$ is considerably closer to $RB{E}_{r}$ than $RB{E}_{m}$ in magnitude.

It should be noted that, as with other RBE models, all endpoints from the MM model are predicting a distal range extension (1–3 mm) of biological effect beyond the absorbed dose distribution. The magnitude of the shift is ordered (smallest - largest): $RB{E}_{r}$, $RB{E}_{r{\rm{\& }}m}$ and $RB{E}_{m}$.

**Figure 2:**
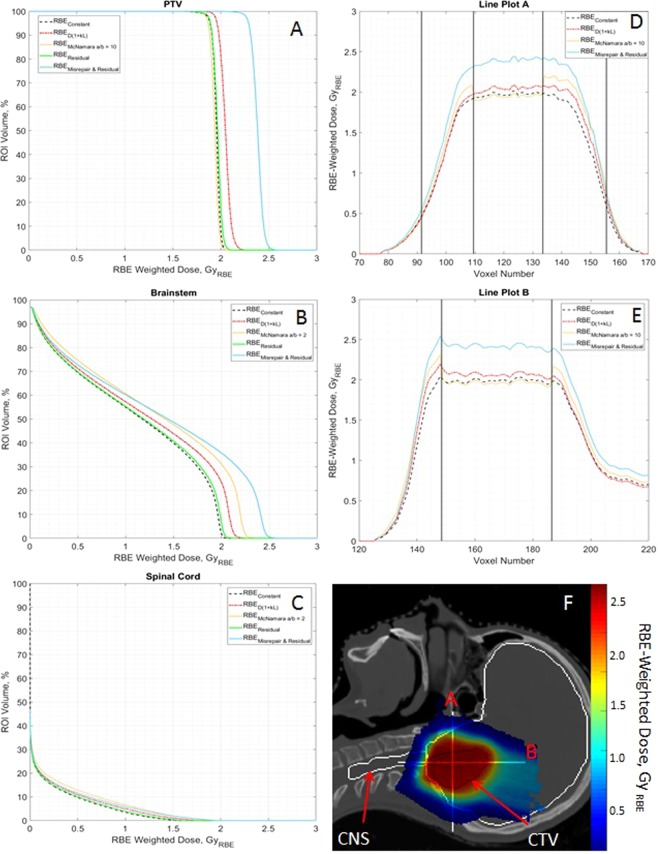
(**A**–**C**) Dose-volume histograms (DVHs) for the planned target volume (PTV) and organs at risk (OARs) for the SFUD ependymoma patient showing RBE-weighted dose using a constant RBE (dashed black), $D(1+\kappa L)$ (dot-dash red), the McNamara model (solid yellow), an endpoint of residual DSB yield (solid green) and an endpoint of combined misrepaired and residual DSB yield (solid blue). $\kappa =0.055\mu m\ {keV}^{-1}$, $\alpha /\beta =2$ Gy for spinal cord and brainstem and $\alpha /\beta =10$ Gy for PTV. (**A**) PTV DVH. (**B**) Brainstem DVH. (**C**) Spinal Cord DVH. (**D**–**F**) Line plots of RBE-weighted dose against voxel number with spatial position in patient anatomy. The same legend is followed for line plots as for DVHs. (**D**) Line plot A. (**E**) Line plot B. (**F**) Line plot positions of (**A**,**B**) are shown on the patient with CNS(white) and CTV (black contours and RBE-weighted dose ($Dos{e}_{r{\rm{\& }}m}$).

Figures 2A–C show the dose volume histograms (DVHs) for the RBE-weighted dose of various RBE models for the PTV, brainstem and spinal cord. These are: constant RBE of 1.1 ($RB{E}_{Constant}$), $RB{E}_{D(1+\kappa L)}$, $RB{E}_{McNamara}$, $RB{E}_{r}$ and $RB{E}_{r{\rm{\& }}m}$. $RB{E}_{m}$ is not shown on the DVHs as the values are much higher than the other models. The effect of $RB{E}_{m}$ can be seen in $RB{E}_{r{\rm{\& }}m}$. An $\alpha$/$\beta$ of 2 Gy has been assumed for the brainstem and spinal cord in the McNamara model.

In Figure 2D–F, the RBE models broadly follow the same shape although there is a substantial difference in magnitude. For the endpoints predicted by the MM model, the patterns are broadly similar across the three ROIs. $RB{E}_{r}$ follows $RB{E}_{Constant}$ closely in the PTV, brainstem and spinal cord while $RB{E}_{r{\rm{\& }}m}$ is consistently larger than the $RB{E}_{Constant}$, leading to predicted higher dose per fraction and total dose.

$RB{E}_{r}$ is in close agreement with the McNamara model for the PTV where both follow $RB{E}_{Constant}$. However, for the different $\alpha$/$\beta$ tissue, in this case assuming 2 Gy for brainstem and spinal cord, the difference is substantial with the McNamara larger at all dose-volume points. A change of $\alpha$/$\beta$ will lead to differences between the MM and McNamara models as only McNamara takes this parameter as an input; the MM model is developed for a generic cell. $RB{E}_{r}$ is either smaller than or equal to the $RB{E}_{D(1+\kappa L)}$ for all ROIs.

$RB{E}_{r{\rm{\& }}m}$ is consistently larger than $RB{E}_{D(1+\kappa L)}$ in all ROIs. However, its relationship with $RB{E}_{Mc}$ has greater complexity. For the PTV, it is much larger than all other models including $RB{E}_{Mc}$, which in this case gives the lowest biological dose. However, this changes for the brainstem and spinal cord where $\alpha$/$\beta$ = 2 Gy. For these ROIs, $RB{E}_{Mc}$ is higher at lower doses before becoming lower than $RB{E}_{r{\rm{\& }}m}$ at the higher doses. This is especially significant for serial organs such as the brainstem and the spinal cord, as the primary clinical concern is the maximum dose. $RB{E}_{r{\rm{\& }}m}$ is consistently greater than the $D(1+\kappa L)$ with the difference between them increasing at higher doses.

Figure 2A–C displays the line plots and their position within the patient anatomy. These plots show similar relationships to the DVHs with additional spatial information. The MM model estimates a greater RBE-weighted dose across the clinical target volume (CTV), central nervous system (CNS) and partially the body. The McNamara model predicts a higher dose in the low dose region surrounding the target. This is due to the combination of both high LET, as the region overlaps with the lateral edges and distal falloff of the beams, and the low $\alpha$/$\beta$ of the body.

It should be noted that the McNamara and LET-weighted dose models use $LE{T}_{d}$ as the averaging method for LET while the MM model uses $LE{T}_{t}$. It is often stated in the literature that $LE{T}_{d}$ has greater biological relevance than $LE{T}_{t}$ although rigorous investigation has not been carried out^32^. Unlike the majority of RBE models, our residual and misrepair correlations use $LE{T}_{t}$ as they derive from DNA level simulations. At this length scale, the non-homogeneous nature of the dose distribution makes $LE{T}_{d}$ very noisy and therefore unsuitable^33^.

## Discussion

In this work, we calculate predicted yields of residual and misrepaired DSBs using the correlations established in previous work by our group^20^. These correlations have been extended to RBE with endpoints of residual and misrepair DSBs. The RBE-weighted dose for these endpoints was subsequently calculated on a representative PBT clinical plan anatomy for the first time and compared to the McNamara model and LET-weighted dose model.

In its present form, the use of the MM model instead of the other models in this paper may have a clinical effect on treatment planning. In the vast majority of voxels, the predicted RBE-weighted dose (for $Dos{e}_{r{\rm{\& }}m}$) is greater than both the McNamara and LET-weighted dose model. In the presented case and cases with similar geometry (critical structure distal to target), this may result in a treatment planner expending more effort in reducing the dose to the brainstem and the PTV-brainstem overlap area in the optimisation process to remain within standard dose protocols. Also, methods used to reduce LET in critical structures, such as avoiding distal fall off into such structures via the alteration of beam angles and beam number^34^, may be applied more strongly. As this constrains the optimisation problem further, it may lead to a degraded absorbed dose distribution.

As previously stated, the MM model finds very high RBE values for misrepair, an endpoint of DNA damage. This means the MM model is predicting that misrepaired DSBs are occurring at a much greater frequency in proton therapy than photon treatments. This difference highlights an issue with phenomenological models, since applying an RBE value resulting from fitting to *in vitro* cell survival data would not be able to distinguish between the different modes of damage which, in some combination, result in cell death or other clinically relevant endpoints such as tissue toxicity.

Furthermore, it is known that RBE varies with endpoint^2^. Thus any potential method to obtain a variable RBE model must be able to predict multiple endpoints for a full account of variable RBE. Existing phenomenological models are intrinsically unable to do so and can only predict a single RBE value. In contrast, mechanistic models, such as the MM model^20^, can provide any number of outputs, by predicting different types of damage and incorporating multiple number of different repair pathways.

While these results show the potential of mechanistic models, there are several steps to be completed before the ultimate aim of clinical application can be realised. It is not currently understood in the literature how and in what proportion residual and misrepaired DSBs lead to the endpoints of cell death or overall tissue toxicity. By comparing $Dos{e}_{r{\rm{\& }}m}$ to $Dos{e}_{Mc}$ and $Dos{e}_{w}$, it is implied combined residual and misrepair yield is equivalent to *in vitro* cell kill. However, it is reasonable to assume the two damage modes have different probabilities of resulting in this endpoint as well as others. Consideration of the resultant chromosome aberrations from both damage modes is required to close the gap and estimate cell death.

The inclusion of chromosome aberrations may also be important in discerning whether the prediction of misrepair can be correlated with carcinogenesis. Several different types of chromosome aberrations, such as dicentric chromosomes, make up the misrepaired DSB yield and have differing survivable probabilities. The risk of carcinogenesis is an important concern in proton therapy, especially when the case mix includes a high proportion of paediatric patients. Therefore, this is a key motivation for the development and clinical implementation of mechanistic models.

In the literature, the RBE models can be broadly separated into two groups, those which keep to the physical parameters of absorbed dose and LET (e.g. LET-weighted dose) and those which add more consideration of biology through $\alpha /\beta$ ratios (e.g. McNamara model). The MM model sits between these two with its consideration of initial DSBs and the consequence of DNA repair for a generalised cell. While there is good evidence for RBE dependence on $\alpha /\beta$, the significant uncertainties in the $\alpha /\beta$ values themselves restrict their use in the clinic. Instead, it would be better to describe the parameters of the different pathways leading to cell death. Only mechanistic models can achieve this and this is one of the future aims of the DaMaRiS model.

In addition, the DaMaRiS repair model is currently restricted to modelling NHEJ, which is considered the dominant DNA repair pathway in human cells^35^. However, another DSB repair pathway, homologous recombination (HR), has a significant impact on repair within human cells in some phases of the cell cycle and is required for a complete understanding of DSB repair. The introduction of HR into the model will lead to, and allow incorporation of, the cell cycle and tissue-specific parameters into the model. This work is currently underway.

Both the incorporation of HR and linking residual and misrepaired DSBs to endpoints with greater clinical relevance are longer-term goals. In the short term, validation of some underlying assumptions in the model is required to improve confidence in its accuracy. Currently, the simulation consists of an irradiated spherical water volume with the resulting energy depositions are then transferred onto the DNA structure with consideration of DNA’s greater density (1.406 g $c{m}^{-3}$). This assumes the difference between proton ionisation cross sections of water and the molecules constituting DNA (guanine, adenine, thymine and cytosine) do not significantly differ. While this is a common assumption made in Geant4 DNA simulations of DNA damage and treatment planning in general, there is evidence demonstrating substantial differences^36^. As the damage proportion of the model has been fit to experimental data the effect of this assumption should be reduced, but further examination is required.

There are several other assumptions which may also require investigation. Firstly, only the DNA damage caused by protons and the secondary electrons created by the primary protons in the nucleus is modelled. The lack of neutrons in the model may affect the ability to predict secondary cancers^1^, a principal aim. Secondly, the predicted misrepair yield has been shown to match other models in the literature but has not yet been directly compared to experimental data.

Furthermore, an assumption which affects most RBE models is the ability of a single averaged LET value to inform on the biological effect at each voxel. At each voxel in one or more beam paths, there is a distribution of particles with differing energy and thus a spectrum of LET values. A single value for LET is obtained by using an averaging method, commonly $LE{T}_{d}$ or $LE{T}_{t}$. Then, two voxels of the same single value of LET and absorbed dose, according to all RBE models discussed in this paper, have the same clinical effect, despite the potential for vast differences in the LET spectra. It is feasible that these differences in spectra can have a differing radiobiological effect of clinical significance.

These issues are challenges to the model, but as these are resolved, the model will provide further insight and draw attention to areas which require further study. We suggest that one of the key advantages of mechanistic models is their ability to make predictions outside of a *posteriori* knowledge for further study.

Clinically, several further steps need to be explored if these models are to be applied in treatment planning. Firstly, further validation work is crucial before any clinical application. Initially, this will include cellular experiments into the mechanical processes of DNA damage, DNA repair and chromosome aberrations, where experimental data in the literature is currently lacking. The next step would be providing the clinician with maps of residual, misrepair or other endpoints for patients as well as investigation of indirect optimisation strategies such as beam angle selection and target segmentation techniques^37^. After this, direct optimisation, where DNA damage parameters are used alongside absorbed dose in the TPS optimiser, could follow. While full optimisation is some distance away due to the considerable validation work required, the concept of ‘no-’ or ‘low-cost’ optimisation, proposed by others^29^, can be achieved in the short term. This optimisation is one which does not cause clinically significant degradation in the absorbed dose distribution and is achievable via multi-field optimisation (MFO) in IMPT. It would also be beneficial to conduct statistical analysis on a range of Christie patients to study any trends in prediction of biological effect.

## Data Availability

The datasets used and generated during and/or analysed during the current study are available from the corresponding author on reasonable request.
